# TNF-α Modulates P-Glycoprotein Expression and Contributes to Cellular Proliferation via Extracellular Vesicles

**DOI:** 10.3390/cells8050500

**Published:** 2019-05-24

**Authors:** Tandressa S. Berguetti, Lucas S. P. Quintaes, Thais Hancio Pereira, Marcela C. Robaina, André L. S. Cruz, Raquel C. Maia, Paloma Silva de Souza

**Affiliations:** 1 Laboratório de Hemato-Oncologia Celular e Molecular, Programa de Hemato-Oncologia Molecular, Instituto Nacional de Câncer (INCA), Rio de Janeiro 20231-050, Brazil; tata.berguetti@hotmail.com (T.S.B.); lucas.s.p.quintaes@gmail.com (L.S.P.Q.); thais.hancio@gmail.com (T.H.P.); mrobaina@ymail.com (M.C.R.); 2 Programa de Pós-Graduação Strictu Sensu em Oncologia, INCA, Rio de Janeiro 20231-050, Brazil; 3 Laboratório de Fisiopatologia, Polo Novo Cavaleiros, Campus UFRJ-Macaé, Universidade Federal do Rio de Janeiro, Rio de Janeiro 21941-909, Brazil; andrecruz@macae.ufrj.br

**Keywords:** P-glycoprotein, ABCB1, TNF-α, extracellular vesicles, microparticles, drug resistance, MDR

## Abstract

P-glycoprotein (Pgp/ABCB1) overexpression is associated with multidrug resistance (MDR) phenotype and, consequently, failure in cancer chemotherapy. However, molecules involved in cell death deregulation may also support MDR. Tumor necrosis factor-alpha (TNF-α) is an important cytokine that may trigger either death or tumor growth. Here, we examined the role of cancer cells in self-maintenance and promotion of cellular malignancy through the transport of Pgp and TNF-α molecules by extracellular vesicles (membrane microparticles (MP)). By using a classical MDR model in vitro, we identified a positive correlation between endogenous TNF-α and Pgp, which possibly favored a non-cytotoxic effect of recombinant TNF-α (rTNF-α). We also found a positive feedback involving rTNF-α incubation and TNF-α regulation. On the other hand, rTNF-α induced a reduction in Pgp expression levels and contributed to a reduced Pgp efflux function. Our results also showed that parental and MDR cells spontaneously released MP containing endogenous TNF-α and Pgp. However, these MP were unable to transfer their content to non-cancer recipient cells. Nevertheless, MP released from parental and MDR cells elevated the proliferation index of non-tumor cells. Collectively, our results suggest that Pgp and endogenous TNF-α positively regulate cancer cell malignancy and contribute to changes in normal cell behavior through MP.

## 1. Introduction

Success of cancer therapy may be affected by several factors driving tumor cells to multidrug resistance (MDR) phenotype—an intrinsic or acquired resistance to several structurally unrelated compounds [1]. Overexpression of P-glycoprotein (Pgp, ATP-binding cassette subfamily B member 1 (ABCB1), multidrug resistance protein 1 (MDR1)), an ATP-binding cassette (ABC) transporter, plays a critical role in the MDR profile pumping anticancer drugs out of cancer cells [2]. Moreover, clinical studies have shown Pgp overexpression in liver, pancreas, kidney, ovarian, gastrointestinal tract tumors and leukemia [3,4]. The relevance of Pgp overexpression in cancer has been associated not only with MDR, but also with drug bioavailability, tumor biology and apoptosis resistance [5,6,7]. 

Studies have shown that extracellular vesicles (membrane microparticles (MP)) released from MDR cells are able to carry Pgp and transfer it to other cells, changing their molecular profile [8,9]. MP are small vesicles—ranging from 0.2 to 1 µM in diameter—which arise from plasma membrane blabbing of normal or cancer cells [10]. It has been discussed that MP or other small vesicles, such as exosomes (nanovesicles ranging from 30–100 nm in diameter, derived from the endosomal pathway), produced by MDR cells may trigger signaling cascades in cancer recipient cells, contributing to drug resistance and cellular survival [9,10,11,12].

The involvement of Pgp in the apoptotic process has been discussed for over 15 years. Studies have demonstrated that MDR leukemic cells become sensitive to tumor necrosis factor (TNF) ligand members after Pgp efflux function blockage [13,14]. It was also shown that Pgp could promote resistance to apoptosis induced by γ-irradiation or growth factor withdrawal [6,15]. Moreover, it has been reported that there is an association between Pgp overexpression and anti-apoptotic proteins supporting MDR maintenance [9]. 

Apoptosis induced by TNF superfamily ligands, which bind to cell surface death receptors, has been a tool explored for therapeutic approach [16,17]. TNF ligands also play an important role in the tumor microenvironment, contributing to tumor progression, invasion and metastasis [18,19]. Specifically, both soluble TNF-α cytokine (sTNF-α), and its membrane-bound precursor protein (tmTNF-α), have biological effects in cancer [20,21]. Furthermore, it was reported that exosomes derived from non-cancer cells can carry endogenous TNF-α, and maintain the CD4^+^ T cell proliferation index in long-term culture [22]. 

In a previous study, using TaqMan Apoptosis Array, our group identified several apoptotic genes associated with Pgp expression. It was precisely demonstrated that endogenous TNF-related apoptosis-inducing ligand (TRAIL) expression (a ligand member of TNF superfamily) is inversely related to Pgp expression. As well as this, TRAIL induced differentiated apoptosis in MDR cells when compared with the parental cell line [23]. Given the association between Pgp and apoptosis-related proteins, and that TNF-α is associated with the cancer microenvironment, we hypothesized that Pgp and TNF-α could contribute to cellular malignancy maintenance and stimulate a phenotype switch in non-cancer cells. Here, we report that recombinant TNF-α regulates endogenous TNF-α and Pgp expression levels in parental and resistant cell lines, which may impact on the MP content released from these cells. Furthermore, TNF-α appears to be favorable for maintenance of cancer cell malignancy and seems to contribute to normal cells’ aberrant proliferation.

## 2. Materials and Methods

### 2.1. Cell Lines and Cell Culture

The parental KB-3-1 cell line (derived from human cervical cancer—HeLa subclone) and the drug-resistant KB-C1 cells (a gift from Dr. Michael Gottesman—Lab of Cell Biology, National Cancer Institute (NCI), National Institutes of Health (NIH), USA) were cultured as previously described [23]. The KB-C1 cells were cultured with colchicine 1 µg/mL (Sigma-Aldrich). The Immortalized human fibroblast (IHF) cell line from ATCC (Bj-5ta—foreskin fibroblast immortalized with hTERT) was cultured in Dulbecco’s modified Eagle’s medium (DMEM) in high glucose (4.5 g/L), supplemented with 1 mM sodium pyruvate, 2 mM L-glutamine, 100 U/mL penicillin/streptomycin and 10% fetal bovine serum (FBS). The cell lines were maintained in a humidified incubator with 5% CO_2_ at 37 °C, and were tested for mycoplasma contamination and short tandem repeat (STR) analysis. 

A total of 1.5 × 10^5^ IHF recipient cells were seeded on a 6-well plate and co-cultured with isolated MP (described below) derived from KB-3-1 (3-1MP) or MP derived from KB-C1 (C1MP) for 24 h. 

### 2.2. Cytotoxicity Assay

The viability of KB-3-1 and KB-C1 cell lines were measured by MTT assay [24]. Briefly, 5 × 10^3^ cells were seeded in each well of a 96-well plate. After 24 h, cells were incubated with cisplatin (Accord), doxorubicin (Bergamo), colchicine or recombinant human TNF-α (rTNF-α) (Sigma-Aldrich—T0157) and further cultured for 24, 48 and 72 h. Then, cells were incubated with MTT reagent in a humidified incubator with 5% CO_2_ at 37 °C for 3 h and solubilized in DMSO. The absorbance was measured at 570 nm in a spectrophotometer (EZ Read 400, Biocrhom).

### 2.3. MP Purification

MP were isolated from 4 × 10^5^ KB-3-1 or KB-C1 cell cultures by differential centrifugation. Here, we optimized the protocol previously described [9]. The cells were incubated with 10 ng/mL rTNF-α for 24 h, and then cell supernatants were centrifuged twice at 2500 *g* (Allegra X-22R, Beckman Coulter) for 10 min each to pellet the whole cell population. Supernatants were further centrifuged at 16.000 *g* (Eppendorf Centrifuge 5415R) for 2 h 30 min to pellet MP. Then, MP were washed in sterile phosphate buffered saline (PBS) and pelleted again. Identification of an annexin-V positive MP population was performed as described earlier [9]. Protein content of isolated MP were performed as descried below. 

### 2.4. Western Blot Analysis

Total cell lysates and MP protein content was carried out as previously described [25]. Protein content was quantified using the BioRad DC protein assay kit, and 30 or 40 µg of total protein were loaded into 7, 10 or 12% acrylamide gels. Proteins were transferred to Hybond-P membranes (GE Healthcare, Buckinghamshire, UK). Anti-Pgp (clone 219, Covance), Anti-TNF-α (D1G2, Cell Signaling), Anti-IκBα (L35A5, Cell Signaling), Anti-Caspase-3 (clone CPP32, BD Biosciences), Anti-Cleaved Caspase-3 (Asp175) (5A1E, Cell Signaling), Anti-p44/42 MAPK (Erk1/2, Cell Signaling), Anti-phospho-Erk1/Erk2 (Thr185, Tyr187, Invitrogen) and Anti-Hsc70 (clone B-6, Santa Cruz Biotech) were diluted at 1:1000 and used for western blot, following incubation with anti-rabbit or anti-mouse secondary antibodies (Sigma-Aldrich, IgG), diluted at 1:30,000. Protein expression was visualized using an ECL Western Blotting Substrate kit according to the manufacturers’ instructions (Western Blotting Analysis System, Amersham Biosciences). The densitometry analysis relates to the pixel densitometry of target bands under respective constitutive bands obtained using software ImageJ (NIH, ImageJ 1.49v, Madison, WI, USA). The ration was normalized by control.

### 2.5. Real Time Quantitative PCR (qRT-PCR)

Total RNA from cell lines was extracted using Trizol reagent (TRIzol, Invitrogen, CA, USA), according to the manufacturers’ instructions. RNA concentration and purity were analyzed by 260/280 nm spectrophotometry using NanoDrop 1000 (Thermo Scientific, Waltham, MA, USA). We used 500 ng RNA to synthesize complementary DNA (cDNA) with SuperScript III First-Strand (Invitrogen, CA, USA). For real-time quantitative PCR (qRT-PCR), the following Taqman probes from Applied Biosystems were utilized: Pgp (*ABCB1* gene) (Hs00184491_m1), and GAPDH (Hs99999905_m1) as an endogenous reference. For the *TNFA* gene, the SYBR Green PCR Master Mix kit (Applied Biosystems, Waltham, MA, USA) was used according to the manufacturers’ instructions. The following *TNFA* primers were utilized: Forward—5′ CAG CCT CTT CTC CCT GA 3′ and Reverse—5′ AGA TGA TCT GAC TGC CTG GG 3′. The 2^−ΔΔCT^ method was employed to quantify the expression levels between treated cells and controls using a 7500 Real-Time PCR System (Applied Biosystems, MA, USA). All PCR assays were done in duplicate.

### 2.6. Apoptosis Detection

To detect apoptosis, 5 × 10^4^ KB-3-1 cells and 5 × 10^4^ KB-C1 cells were seeded and then incubated with 10, 15, 20 and 30 ng/mL rTNF-α for 24 and 48 h. Following this, the cell lines were blocked with 2% PBS/Bovine Serum Albumin (BSA) for 40 min and submitted to the annexin-V/Propidium Iodide (PI) assay according to the manufacturers’ instructions (Alexa Fluor 488 Annexin V/Dead Cell Apoptosis Kit, Invitrogen). The apoptotic index was analyzed by flow cytometry (FACSCalibur, Becton Dickinson and Company), considering double negative as viable cells, annexin-V staining as initial apoptosis and double positive as late apoptosis/necroptosis.

### 2.7. Detection of Pgp by Flow Cytometer 

To detect Pgp cell surface expression, 5 × 10^5^ KB-C1 cells were seeded and then incubated with 10 and 15 ng/mL rTNF-α for 24 h. Following this, cells were blocked with 1% PBS/BSA for 15 min, washed and incubated with 1 µg anti-P-glycoprotein antibody conjugated with phycoerythrin (UIC2-PE, Immunotech) for 30 min at 37 °C. After washing with 1% PBS/BSA, cells were analyzed by flow cytometer (FACS Calibur, Becton Dickinson and Company). KB-C1 cells with no labeling (autofluorescence) were used as negative control.

### 2.8. UIC2 Shift Assay

The UIC2 shift assay was performed as previously described [26]. Briefly, 5 × 10^4^ KB-C1 cells were seeded and incubated for 24 h with 10 or 15 ng/mL rTNF-α. Following this, cells were incubated for 5 min with 20 μM cyclosporine A (CsA) (Sandoz) at 37 °C. KB-C1 cells were then incubated with 2 μg/mL UIC2-PE (Immunotech) for 30 min at 37 °C. After that, cells were washed and then analyzed by flow cytometry (FACS Calibur, Becton Dickinson and Company). The UIC2 shift was defined as the difference between antibody binding in the presence or absence of CsA. 

### 2.9. Efflux Activity of Pgp by Flow Cytometer

To analyze Pgp efflux activity, 1 × 10^6^ cells were incubated with 200 ng/mL of Pgp substrate rhodamine 123 (Sigma-Aldrich) or calcein-AM (a gift from Dr. João Viola—Progra, of Immunology and Tumor Biology, Instituto Nacional de Câncer, Brazil) (C3100MP, Life technologies) in the presence or absence of the Pgp inhibitor CsA (20 µM) for 45 min at 37 °C. After washing, cells were incubated in the presence or absence of CsA for 45 min and then analyzed by flow cytometry (FACS Calibur, Becton Dickinson and Company). 

To inhibit Pgp function, KB-C1 cells were incubated with 10 µM CsA in the presence or absence of rTNF-α for 24 h. The cells were analyzed by flow cytometry for efflux activity and using qRT-PCR for messenger RNA (mRNA) expression levels of *ABCB1* and *TNFA*.

### 2.10. Enzime-Linked Immunosorbent Assay (ELISA)

KB-3-1 and KB-C1 cells were incubated with rTNF-α for 30 min (after this time, cells were washed and cultured for 24 h) or 24 h, and then cell-free supernatants were assessed for TNF-α protein levels using human TNF alpha ELISA Ready-SET-Go kit (Affymetrix). Also, cell-free IHF supernatants were assessed after co-culture with MP derived from KB-3-1 cells (3-1MP) or MP derived from KB-C1 cells (C1MP) for 24 h.

### 2.11. Anchorage Independent Growth Assay

For the anchorage independent growth assay, 5 × 10^4^ IHF cells were seeded and co-cultured with 3-1MP or C1MP for 48 h in each well of a 24-well plate. 3-1MP or C1MP were added daily. Following incubation, cells were tripsinized and 5 × 10^3^ cells were resuspended in a medium containing MP and 0.6% agarose. The cells were seeded in duplicates in 6-well plates coated with 1.2% agarose-supplemented growth medium to prevent cell adhesion. After being plated, colonies were analyzed after 18 days in culture. Images were captured using Axio Observer.Z1 microscopy (Zeiss) under 20× magnification. Colony diameter was obtained measuring image pixels with the aid of a stage micrometer slide.

### 2.12. Wound Healing Assay

For the wound healing assay, 5 × 10^4^ IHF cells were seeded in duplicates in a 6-well plate per well. After cell adhesion, wounds were generated by scratching the adhesion surface with a sterile tip. Cells were then washed with PBS and co-cultured with 3-1MP or C1MP in DMEM 0.1% FBS. The images were captured immediately after co-culture (0 h) and after 16 h, using an Axio Observer.Z1 microscope (Zeiss) under 10× magnification. The migration rate was evaluated by the area ratio between scratch lines in 16 h and 0 h, using the image software ImageJ (NIH, ImageJ 1.49v, Madison, WI, USA).

### 2.13. Crystal Violet Incorporation Assay

For proliferation assay, 8 × 10^2^ IHF cells were seeded per well in triplicates in 96-well plates and 3-1MP or C1MP were added daily. After 16 h, cells were fixed with ethanol for 10 min, washed with PBS and stained with 0.05% crystal violet in 20% ethanol for 10 min. Cells were then solubilized with methanol and read on a spectrophotometer at 595 nm (EZ Read 400, Biocrhom) at the indicated times. 

### 2.14. Statistical Analysis

The statistical analyses were performed using the Student’s *t*-test, the Mann–Whitney test, one-way ANOVA with Dunn’s’ posttest or two-way ANOVA with Bonferroni posttest analysis of Graph Pad Prism 5.0 software. A value of *p* < 0.05 was considered statistically significant.

## 3. Results

### 3.1. rTNF-α Induces Differential Effects on MDR Cells

**Figure 1:**
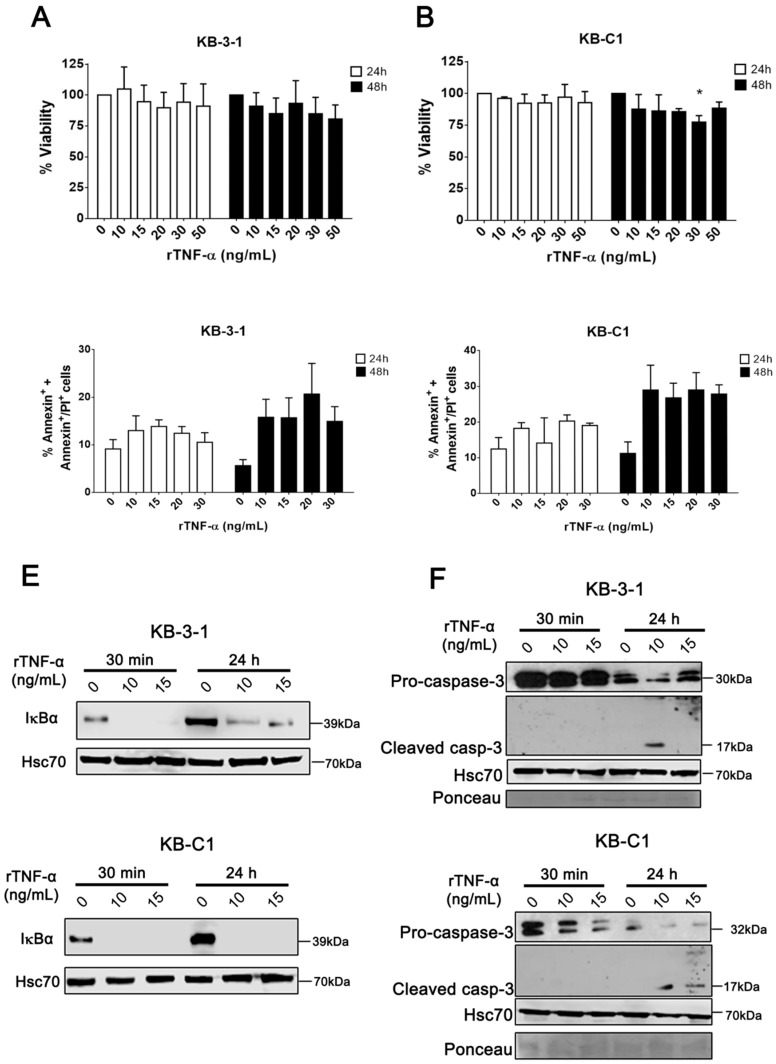
Analysis of parental and multidrug resistance (MDR) cell viability after recombinant human tumor necrosis factor-alpha (rTNF-α) treatment. Cell viability determined by (**A**,**B**) MTT assay and (**C**,**D**) annexin-V positive staining after 24 or 48 h of rTNF-α treatment in different concentrations. Immunoblot analysis of (**E**) IκBα, (**F**) pro-caspase-3 and cleaved caspase-3 protein levels determined after 30 minutes or 24 h of 10 or 15 ng/mL rTNF-α treatment. Data are means ± SD of three independent experiments. One-way ANOVA with Dunn’s posttest and *t*-tests were used to analyze the MTT assay and annexin-V staining results, respectively. * *p* < 0.05 indicates the statistical significance of data compared to non-treated cells.

In order to study the MDR phenotype under rTNF-α effect, we utilized a well-characterized model of a resistant, Pgp-positive cell line [27] (Supplementary Figures S1 and S2). Considering that TNF-α is a cytokine that can induce apoptosis [28], we first evaluated the effect of rTNF-α on cell viability in resistant and parental cell lines. For this, both cell lines were incubated for 24 or 48 h with 0, 10, 15, 20, 30 and 50 ng/mL of rTNF-α. According to our data, rTNF-α induced no changes on cell viability in neither parental nor MDR cell lines after 24 h of incubation (Figure 1A,B). However, 30 ng/mL of rTNF-α reduced KB-C1 cell viability with a statistically significant *p* value after 48 h, though we could not observe changes in cell death by annexin-V/PI analysis (Figure 1C,D; Supplementary Figure S3). 

The soluble TNF-α (sTNF-α) can interact with cells through specific receptors, TNFR1 or TNFR2, and trigger downstream signaling, such as the NFκB pathway [29]. Here, we confirmed that 10 and 15 ng/mL of rTNF-α, with incubation times of 30 min or 24 h, induced IκBα degradation, suggesting NFκB pathway activation in resistant and sensitive cell lines (Figure 1E). This result also confirms the proper functionality of rTNF-α in both cell lines. Following this, using 10 and 15 ng/mL of rTNF-α for 30 min or 24 h, we evaluated the caspase-3 expression levels in resistant and parental cell lines. Our data revealed higher levels of reduced pro-caspase-3, followed by cleaved caspase-3, in KB-C1 cells, and a slightly reduction of pro-caspase-3 in KB-3-1 cells (Figure 1F). By phase contrast microscopy, we could not observe changes in cellular/nuclear morphology after rTNF-α treatment in both cell lines (data not shown). Collectively, these data suggest that rTNF-α induced a differential and possibly non-cytotoxic response in parental and MDR cell lines, since rTNF-α did not trigger apoptosis in KB-C1 cells. 

### 3.2. rTNF-α Modulates Pgp and Endogenous TNF-α Expression Levels in Cancer Cells

**Figure 2:**
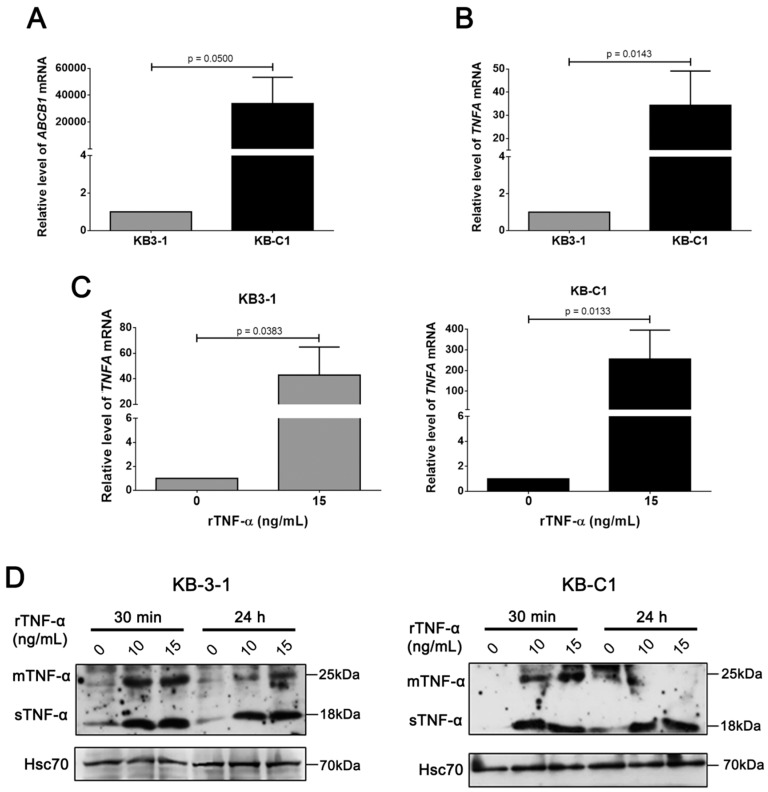
Endogenous TNF-α expression levels in parental and MDR cell lines after recombinant TNF-α treatment. Comparison between KB-3-1 and KB-C1 cells in (**A**) *ABCB1* and (**B**) *TNFA* mRNA levels determined by real time quantitative PCR (qRT-PCR). (**C**) *TNFA* mRNA levels after 24 h of 15 ng/mL rTNF-α treatment determined by qRT-PCR. (**D**) Immunoblot analysis of endogenous TNF-α protein levels after 30 minutes or 24 h of 10 or 15 ng/mL rTNF-α treatment (transmembrane—tmTNF-α; soluble—sTNF-α forms). * *p* < 0.05 indicates the statistical significance of data compared with non-treated cells. Data are means ± SD of three independent experiments. * *p* < 0.05; ** *p* < 0.01 indicates the statistical significance of data compared with parental or non-treated cells. *t*-test was used to analyze the qRT-PCR results.

By using PCR array, we previously identified several apoptotic genes which were differentially expressed in Pgp-positive cells, including TNF superfamily genes [23]. Here, we specifically evaluated the relative expression levels of endogenous TNF-α in a MDR cell line. Initially, we confirmed the higher *ABCB1* expression level in KB-C1 cells (Figure 2A). Next, our data revealed a statistically significant increase (*p* < 0.05) of endogenous TNF-α transcript levels in KB-C1 cells when compared with parental KB-3-1 cells (Figure 2B), indicating a positive correlation between Pgp and TNF-α.

We further analyzed mRNA and protein expression levels of endogenous TNF-α in KB-3-1 and KB-C1 cell lines under rTNF-α treatment. According to our data, rTNF-α induced upregulation of endogenous TNF-α transcript levels in both resistant and parental cell lines after rTNF-α 24 h incubation (Figure 2C). The sTNF-α protein levels also increased in KB-3-1 and KB-C1 cell lines, compared with the untreated condition. However, transmembrane TNF-α (tmTNF-α) protein levels were specifically increased in KB-3-1 cells, while a slightly increase in tmTNF-α protein levels in KB-C1 cells were only observed at earlier times of rTNF-α incubation (Figure 2D). These data suggest a positive feedback involving rTNF-α incubation and activation of *TNFA* gene expression, mostly in the MDR cell line. 

**Figure 3:**
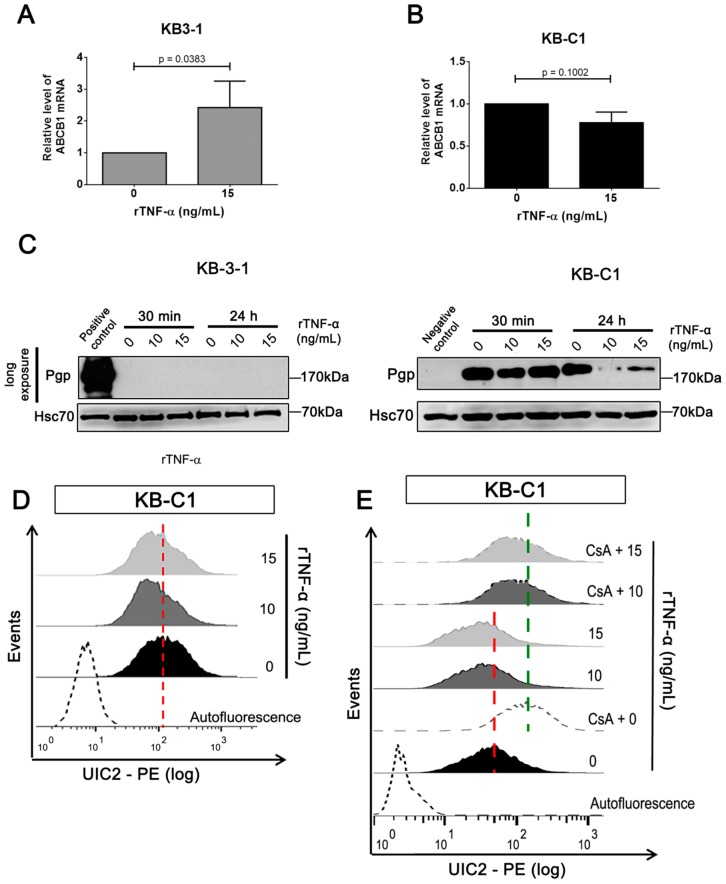
P-glycoprotein (Pgp) expression levels analysis in parental and MDR cell lines after recombinant TNF-α treatment. *ABCB1* mRNA levels determined by qRT-PCR after 24 h of 15 ng/mL rTNF-α treatment in (**A**) KB-31 and (**B**) KB-C1 cells. (**C**) Immunoblot analysis of Pgp protein levels after 30 minutes or 24 h of 10 or 15 ng/mL rTNF-α treatment. Pgp protein levels expression determined by (**D**) specific antibody staining and (**E**) shift-assay after 24 h of 10 or 15 ng/mL rTNF-α treatment in KB-C1 cells, using flow cytometry. Cyclosporine A (CsA) means treatment with 20 µM of cyclosporine A. (**C**) KB-3-1 and KB-C1 cell lines were used as negative and positive controls, respectively. (**D**) Empty histogram represents cellular autofluorescence of KB-C1 cells; black histogram represents non-treated cells; dark gray and light gray histograms represent treatment with 10 or 15 ng/mL rTNF-α, respectively. (**E**) Empty black histogram represents cellular autofluorescence; black histogram represents non-treated cells; empty gray histogram represents treatment with CsA; dark gray and light gray histograms represent treatment with 10 or 15 ng/mL rTNF-α, respectively. Dark gray dashed and light gray dashed histograms represent co-treatment with CsA plus 10 or 15 ng/mL rTNF-α, respectively. Data are means ± SD of three independent experiments. *t*-test was used to analyze the qRT-PCR results. * *p* < 0.05 indicates the statistical significance of data compared to non-treated cells.

We next investigated the effect of rTNF-α on Pgp mRNA and protein expression levels. Our results showed that rTNF-α induced a statically significant increase (*p* < 0.05) in mRNA levels of Pgp (*ABCB1*) in KB-3-1 cells after 24 h, though we could not observe detectable protein levels in these cells (Figure 3A,C (left panel)). This suggests a modified epigenetic profile in these cells, which may impair Pgp translation. Interestingly, ABCB1 transcript levels remained unchanged in resistant cells treated with rTNF-α (Figure 3B). However, rTNF-α promoted expressive reduction on total Pgp protein levels and a slightly decrease in cell surface-Pgp (Figure 3C (right panel); Figure 3D). By UIC2 shift assay, we validated the reduction of Pgp expression levels on the KB-C1 cell surface after 24 h of rTNF-α incubation (Figure 3E). These experiments imply that rTNF-α differentially regulates Pgp expression levels in both parental and MDR cell lines.

### 3.3. Endogenous TNF-α is Upregulated in MDR Cells Independently of Pgp Efflux Activity

**Figure 4:**
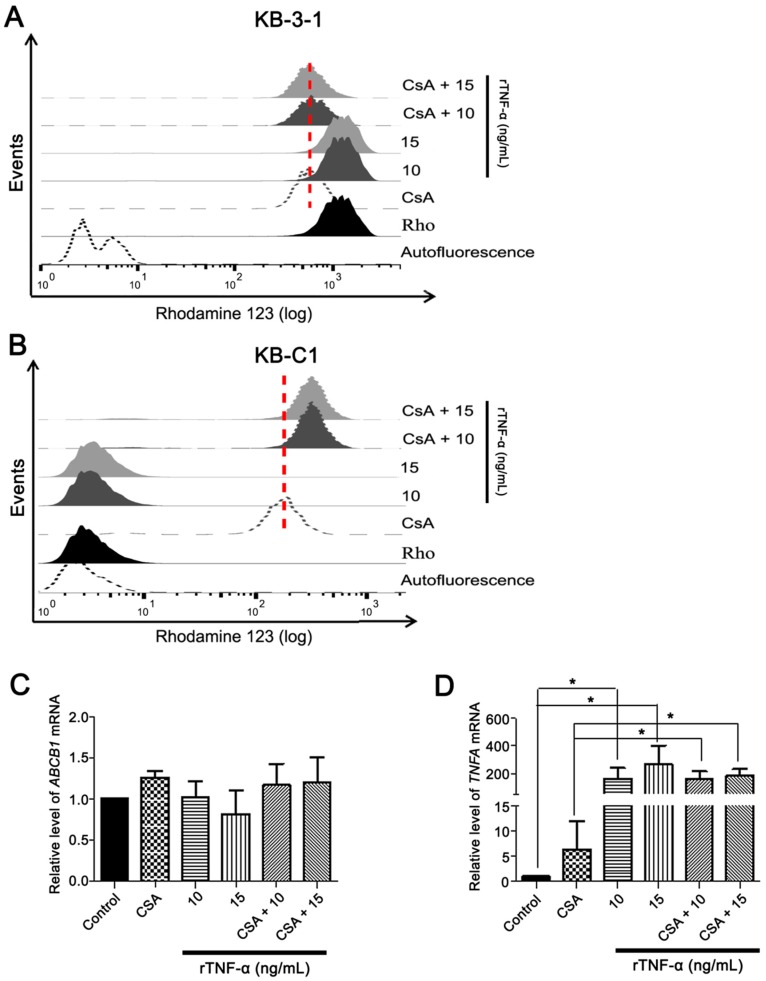
P-glycoprotein (Pgp) efflux activity influence in *ABCB1* and *TNFA* expression in the MDR cell line. (**A**) Efflux activity determined by rhodamine 123 (Rho) assay in KB-3-1 cells after 24 h of rTNF-α treatment, using flow cytometry. (**B**) Pgp efflux activity determined by rhodamine 123 (Rho) assay in KB-C1 cells after 24 h of rTNF-α treatment, using flow cytometry. (**C**) *ABCB1* and (**D**) *TNFA* mRNA levels determined by qRT-PCR after modulation of Pgp efflux activity using cyclosporine A (CsA) and treatment with 10 or 15 ng/mL rTNF-α for 24 h in KB-C1 cells. (**A**,**B**) Empty black histogram represents cellular autofluorescence; black histogram represents Rho staining; empty gray histogram represents Rho staining after CsA incubation; dark gray and light grays histograms represent Rho staining after treatment with 10 or 15 ng/mL rTNF-α, respectively. Dark gray dashed and light gray dashed histograms represent Rho staining after treatment with 10 or 15 ng/mL rTNF-α and CsA incubation, respectively. Data are means ± SD of three independent experiments. One-way ANOVA with Dunn’s posttest were used to analyze the qRT-PCR results. * *p* < 0.05 indicates the statistical significance of data compared to non-treated or CsA treated cells.

The main role of Pgp in a drug-resistance phenotype is associated with its canonic membrane pump function, capable of extruding several chemotherapeutic drugs out of cancer cells [30]. Since rTNF-α induced a reduction in Pgp protein expression levels of the MDR cell line (Figure 3), we further investigated the impact of rTNF-α on Pgp efflux activity in KB-C1 cells. According to our results, blocking Pgp activity with CsA in the presence of rTNF-α reduced rhodamine 123 (Rho) efflux (Figure 4B), but not calcein-AM efflux (Supplementary Figure S4). Aside from this, this experiment demonstrated that rTNF-α could not block or modulate Pgp activity by itself. The efflux activity in KB-3-1 cells under rTNF-α treatment did not show any changes in Rho accumulation (Figure 4A). Interestingly, Pgp blockage did not interfere on *ABCB1* transcript levels, even in the presence of rTNF-α (Figure 4C). However, our data showed that rTNF-α induced mRNA upregulation of endogenous TNF-α, even with a non-functional Pgp (Figure 4D). Collectively, these results demonstrated that rTNF-α positively regulates endogenous TNF-α expression levels independently of Pgp efflux activity.

### 3.4. Cancer Cell-Derived MP Carry Pgp and Endogenous TNF-α and Promote Proliferation in Non-Tumor Cell Lines

**Figure 5:**
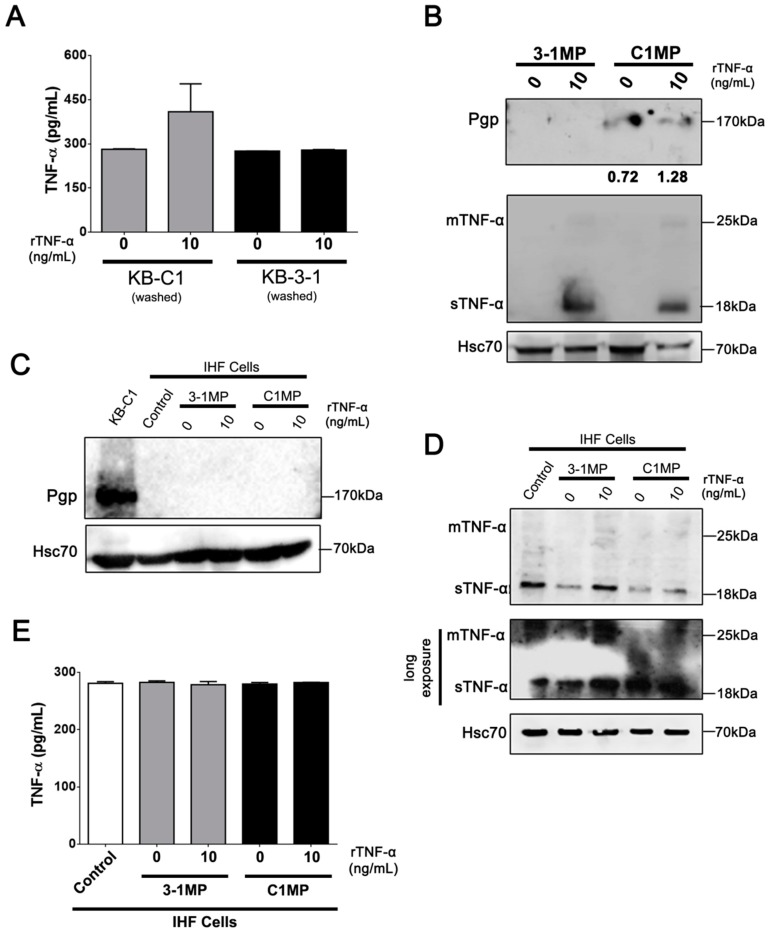
Analysis of microparticles (MP) content derived from parental and MDR cells and its effects in the IHF cell line. (**A**) Comparison of soluble TNF-α secretion in supernatant of KB-3-1 and KB-C1 cells treated for 24 h with 10 ng/mL rTNF-α, determined by ELISA. (**B**) Immunoblot analysis of Pgp and endogenous TNF-α protein levels in 3-1MP and C1MP derived from non-treated or 10ng/mL rTNF-α treated cells. Western blot bands were quantified by densitometry. Immunoblot analysis of (**C**) Pgp and (**D**) TNF-α protein levels in IHF cells after 24 h of incubation with 3-1MP or C1MP derived from non-treated or 10 ng/mL rTNF-α treated cells. (**E**) Comparison of soluble TNF-α liberation in supernatant of IHF cells treated for 24 h with 3-1MP or C1MP derived from non-treated or 10 ng/mL rTNF-α treated cells, determined by ELISA. Data are means ± SD of two independent experiments. One-way ANOVA with Dunn’s posttest post were used to analyze the ELISA results. (**A**) * *p* value = 0.406. MP derived from KB-3-1 (3-1MP); MP derived from KB-C1 (C1MP) cell lines.

rTNF-α modulates Pgp and endogenous TNF-α expression levels in parental and MDR cell lines. Accordingly, we tested whether a TNF-α protein could be secreted in a cultured medium. For this purpose, KB-3-1 and KB-C1 cell lines were incubated with rTNF-α for 30 min, washed, and cultured for 24 h for later measurement of secreted TNF-α. The results revealed no statistical difference in secreted TNF-α protein levels in MDR cells under rTNF-α incubation. However, we observed a tendency towards increased TNF-α secretion in treated KB-3-1 cells (Figure 5A). These results suggest that accumulation of endogenous TNF-α from KB-C1 cells might be delivered to other routes instead of being secreted in soluble extracellular form. 

We previously demonstrated that Pgp and apoptotic proteins can be transported in secreted MP from MDR cells to other cancer cells [9]. Here, we evaluated the protein content of MP released from parental (3-1MP) and MDR (C1MP) cell lines. Initially, the results demonstrated that both cell lines were able to spontaneously release MP into in vitro culture conditions (Supplementary Figure S5). Following this, our results revealed that only 3-1MP and C1MP secreted from rTNF-α treated cells were able to transport sTNF-α and tmTNF-α. In addition, we identified Pgp in MP released exclusively from KB-C1 cells, independently of treatment (Figure 5B). The next step was to analyze whether 3-1MP and C1MP protein content is transferred to a non-cancer cell line. For this purpose, we cultured IHF cells (recipient cells) for 24 h with MP derived from KB-3-1 and KB-C1 cell lines treated with rTNF-α. According to our data, we did not detect Pgp expression levels in non-tumor cells (Figure 5C), which may suggest that IHF cells could not uptake Pgp through C1MP. On the other hand, our results showed high basal levels of sTNF-α protein in IHF cells, while co-culture of IHF with MP released from either parental or MDR cells reduced sTNF-α protein levels in these recipient cells (Figure 5D). Collectively, these results suggest that there is an incompatibility between MP content release from KB-3-1 and KB-C1 cell lines and IHF cells uptake. Aside from this, we also observed no changes on secreted TNF-α protein levels in recipient cells under culturing with 3-1MP and C1MP (Figure 5E).

**Figure 6:**
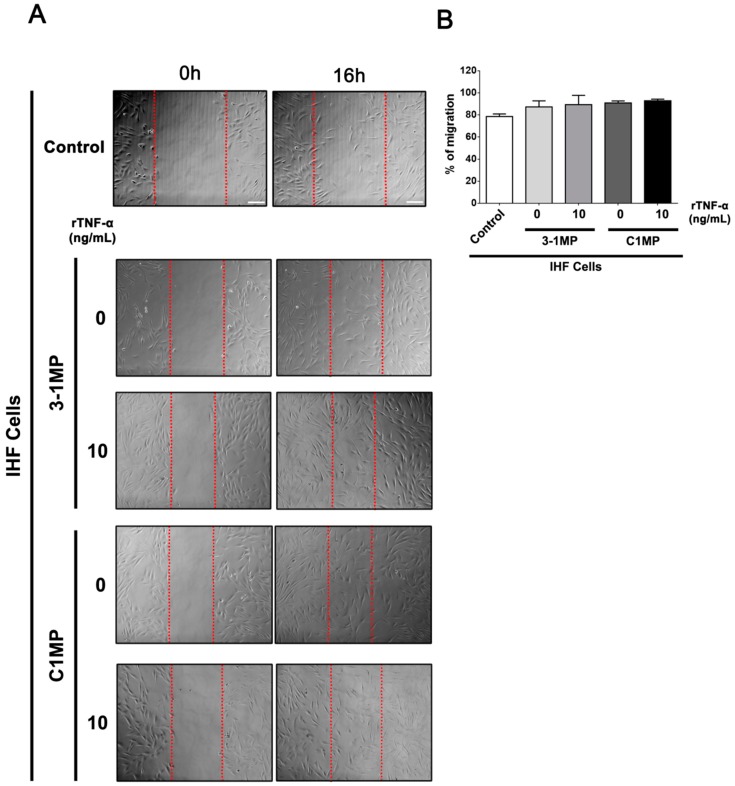
Analysis of IHF cell line migration after incubation with (microparticles (MP) derived from parental and MDR cell lines. IHF cells were scratched and immediately co-cultured with 3-1MP or C1MP secreted by rTNF-α treated tumor cells for 16 h. (**A**) Representative phase contrast images of scratched cells captured at 0 and 16 h time points. Dashed lines represent the limit of analyzed considered area. (**B**) Percentage of IHF cells migration under each condition after 16 h of treatment. Images were acquired using an Axio Observer.Z1, Zeiss, under 10× magnification. Scale bar = 50 μm. Data are means ± SD of three independent experiments. One-way ANOVA with Dunn’s posttest and *t*-tests were used to analyze the wound-healing results.

**Figure 7:**
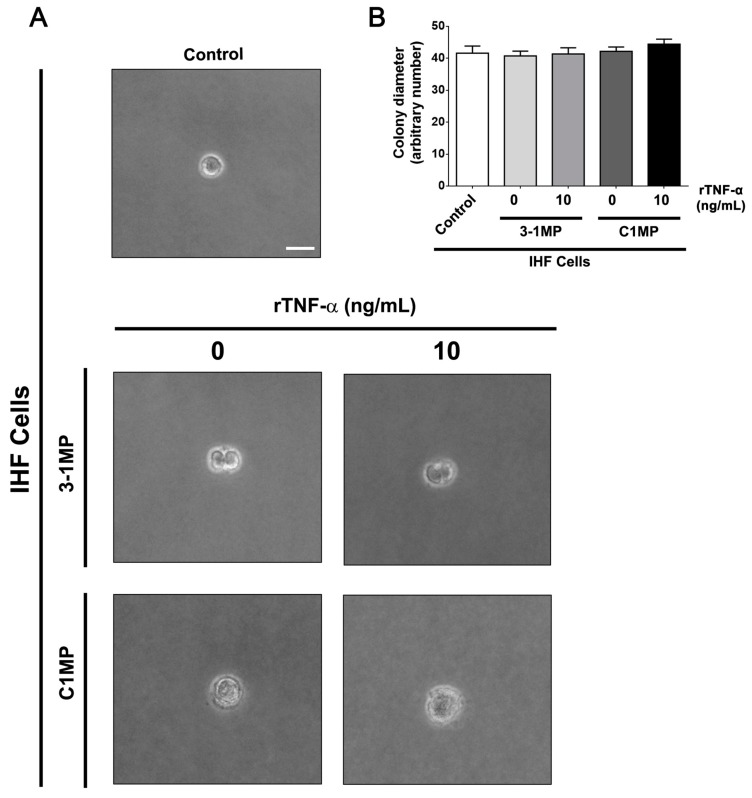
Analysis of IHF colony formation after incubation with microparticles (MP) derived from parental and MDR cell lines. Semisolid-medium growth assay. Following co-culture with 3-1MP or C1MP, IHF cells were cultured in DMEM containing 0.6% agarose. After 18 days, (**A**) colonies were visualized by phase-contrast microscopy and (**B**) colonies’ diameter was measured. (**A**) Representative images of IHF colonies under each condition. Images were acquired using an Axio Observer.Z1, Zeiss, under 20× magnification. Scale bar = 20 μm. Data are means ± SD of three independent experiments. One-way ANOVA with Dunn’s posttest and *t*-tests were used to analyze colony formation assay results.

**Figure 8:**
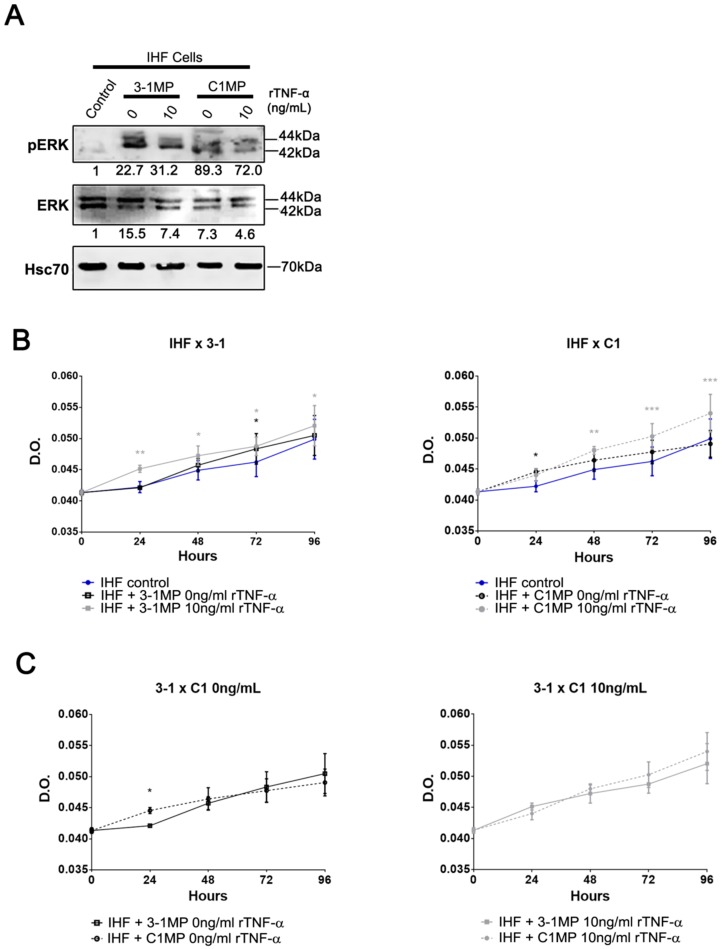
Proliferation rate of IHF cells after incubation with MP (microparticles) derived from parental and MDR cell lines. (**A**) Immunoblot analysis of ERK and phospho-ERK protein levels in IHF cells after incubation with MP derived from non-treated or 10 ng/mL rTNF-α treated KB-3-1 and KB-C1 cells. Western blot bands were quantified by densitometry. (**B**,**C**) Proliferation rate determined by crystal violet incorporation assay during 96 hour treatment with 3-1MP or C1MP derived from non-treated or 10ng/mL rTNF-α treated cells. Data are means ± SD of three independent experiments. Two-way ANOVA with Bonferroni posttest were used to analyze the crystal violet incorporation assay results. Black and gray asterisks represent statistical significance between MP derived from non-treated and rTNF-α-treated cells, respectively. * *p* < 0.05; ** *p* < 0.01; *** *p* < 0.005 indicate the statistical significance of data compared with non-treated cells or IHF incubated with 3-1MP.

Since MP released from cancer cells are able to switch cellular behavior of non-tumor recipient cells [31], we assessed, finally, the influence of MP released from parental and MDR cells on IHF cells. At first, we tested IHF cells’ migratory behavior in vitro. To this end, the monolayer of IHF cells was scratched, and then the cells were immediately co-cultured with 3-1MP and C1MP for 16 h. Our results showed no statistically significant *p* value in IHF cells’ migration status under the different conditions (Figure 6). To further analyze the cell-transforming potential of 3-1MP and C1MP, IHF cells were plated in a non-solid substratum to avoid cell adhesion. No changes were observed in colony sizes; however, morphological observations may indicate a potential growth profile in IHF cells cultured with tumor-derived MP (Figure 7). Following this main interrogation, our results revealed that both 3-1MP and C1MP induced ERK phosphorylation in IHF cells (Figure 8A). In addition, proliferation analysis showed that 3-1MP derived from parental cells treated with rTNF-α stimulated IHF cell growth when compared with the control (Figure 8B (left panel)). By comparison, we also observed that C1MP derived from treated cells induced IHF cell growth at early times (Figure 8B (right panel)). Lastly, our data showed, also in earlier times, that C1MP derived from untreated KB-C1 cells induced higher IHF proliferation in comparison with 3-1MP secreted from KB-3-1 cells (Figure 8C (left panel)). Collectively, these results suggest that MP released from tumor cells previously treated with rTNF-α stimulate IHF cells proliferation, probably via ERK activation. 

## 4. Discussion

Pgp expression, which is encoded by the *ABCB1* gene, is closely related to cancer chemotherapy resistance. Additionally, growing evidence indicates that MDR results from different deregulated cellular mechanisms [32,33]. Pgp expression has also been related to apoptosis signaling pathways [34,35], where an association between Pgp/ABCB1 and apoptotic genes has been shown. We have specifically demonstrated that Pgp regulates TRAIL (ligand member of TNF superfamily) expression and, consequently, TRAIL-induced apoptosis in MDR cells [23]. However, little is known about the association of Pgp and TNF-α, and their impact on tumor phenotype. In this study, we demonstrated that rTNF-α regulates Pgp and endogenous TNF-α expression in cancer cells. In addition, rTNF-α contributes to Pgp and TNF-α release into MP, which consequently regulates cell malignancy. Our results showed that rTNF-α did not induce apoptosis in parental nor MDR cells, not even through pro-caspase-3 reduction levels (Figure 2). These data are supported by Galski and colleagues [36], who showed no apoptosis mediated by TNF-α in either Pgp-positive or Pgp-negative cells. Classically, caspase-3 function is associated with apoptosis activation under both intrinsic factors or extrinsic stimulation, such as DNA damage or TNF-α and TRAIL ligands, being a protease that cleaves specific aspartic acid residues [37,38]. However, studies have demonstrated a non-apoptotic cellular function for caspase-3. In fact, by using an in vitro Casp3^-/-^ model, it was described that caspase-3 influences cell morphology and motility independently of its catalytic activity [39]. Nevertheless, the proteolytic function of caspase-3 is essential for cell death induction [40] and cell differentiation [41]. In that study, the authors showed that caspase-3 inhibition impairs DNA strand-break formation by not activating the caspase-activated DNAse (CAD), an important transient process for myoblast differentiation [41]. Along the same lines, the non-apoptotic function of caspase-3 in myeloid cell differentiation was recently reviewed [42]. Given this evidence, we suggest that rTNF-α might induce a non-apoptotic function of caspase-3 in Pgp-positive cells, as well as inducing an underplayed cytotoxic effect in Pgp-negative cells. 

The expression of Pgp in different tumor types is a concern in cancer treatment [28,43]. The analysis of Pgp expression in metastasis and original osteosarcoma samples from 70 patients demonstrated that Pgp expression could be considered an important prognostic factor [44]. As well as this, a higher chance of relapse was observed in ovary cancer patients that present increased expression of Pgp, suggesting that Pgp expression could be used as a predictive factor [45]. Furthermore, Pgp expression was observed in the late phase of chronic myeloid leukemia samples, but not in the early phase, and was related to survivin expression, an anti-apoptotic protein. These suggest a role in the late phase of disease and apoptosis evasion [46]. 

The role of Pgp expression in apoptosis evasion is associated with an increase of anti-apoptotic proteins, such as different inhibitors of apoptosis proteins (IAP) and TNF family members [23,34,35]. The presence of inflammatory cytokines in a high concentration in a tumor microenvironment has been widely studied to support cancer development and metastasis [47,48]. A case-in-point is TNF-α, which plays a dual role in the cancer context. Despite being described as a tumor necrosis factor, it is known that this molecule is able to support tumor development and invasion [18,19,49]. The development of drug-induced MDR cell lineages induced changes in gene expression profiles in several models [23,50,51]. Here, we show higher mRNA expression levels of *TNFA* in KB-C1 cells in comparison with parental cells, which revealed a positive correlation between *ABCB1* mRNA and *TNFA* (Figure 2B). This result is supported by a previous study [23], which showed the upregulation of apoptosis-related genes in different Pgp-positive cell models. We also show that rTNF-α treatment promotes changes in Pgp/*ABCB1* expression levels in both parental and resistant cell lines (Figure 3). In agreement with our findings, studies have shown that the TNF superfamily ligands regulate Pgp expression in vitro and in vivo [52,53]. Alongside this, it was revealed that long-term TNF-α treatment reduces the Pgp/*ABCB1* transcript and protein levels in intrinsically Pgp-positive colon cancer cells, promoting sensitivity to chemotherapeutic drugs and, possibly, MDR reversal. The authors also observed that TNF-α-activated NFκB required both IκBα and IκBβ degradation for Pgp downregulation [54]. In contrast, other studies have shown Pgp upregulation mediated by TNF-α in tumor and normal cells [55,56,57]. Here, we show that rTNF-α induces an increase in *ABCB1* transcript levels in parental cells without inducing protein expression levels, suggesting that KB-3-1 cells may become resistant after long-term TNF-α stimulation. In MDR cells, rTNF-α promoted a decrease in Pgp expression levels, which may suggest a progressive reduction of drug-resistance profile (Figure 3). Together, our results support the notion that TNF-α regulates Pgp expression differently, depending on the cell phenotype. 

The other point of view regards endogenous TNF-α regulation. Here, we demonstrated that endogenous TNF-α is upregulated under rTNF-α stimulation in both parental and MDR cell lines (Figure 2). It is well-established that treatment with rTNF-α induces a positive feedback upregulation of TNF-α via the NFκB pathway in both in vitro and in vivo models. However, authors often show TNF-α upregulation only in transcripts levels or through secretion of its soluble form [58,59,60]. Our results showed upregulation in soluble, transmembrane and secreted TNF-α in KB-3-1 cells under rTNF-α treatment. In contrast, while being able to induce an increase TNF-α transcript levels and accumulation of sTNF-α, rTNF-α only triggered tmTNF-α enhancement upon short-term treatment in KB-C1 cells (Figure 2D and Figure 5A). In fact, most studies discuss the role of sTNF-α in carcinogenesis [61,62], while tmTNF-α has been pointed out as a resistance modulator in different cancer types [63,64,65,66,67]. Studies with primary breast cancer cells and a xenograft mouse model revealed that cases with high expression of tmTNF-α do not respond to doxorubicin incubation, whereas tmTNF-α knockdown sensitizes breast cancer cells to drug-induced cytotoxicity [66]. Furthermore, high tmTNF-α expression was associated with poor prognosis in colorectal cancer, and in vitro data showed a low cytotoxic effect of 5-fluoroacil in high positive tmTNF-α-expressing cells [67]. Still, in this regard, tmTNF-α knockdown in acute leukemia cells revealed an increase in proapoptotic regulator proteins, as well as in negative cell cycle regulators, indicating a role for tmTNF-α in apoptosis resistance and cell cycle progression [65]. Nevertheless, the differential effects of tmTNF-α in sensitive or MDR cells remain unknown. Together, our results raise the hypothesis that rTNF-α may induce a long-term resistance profile in KB-3-1 sensitive cells via an increase of Pgp and tmTNF-α expression levels, and might promote a long-term chemotherapeutic drug sensitivity in Pgp-positive cells. 

In breast cancer patient samples, TNF-α serum concentration was related to poor prognostic and metastasis development. Although TNF-α function is not associated with hormone receptor expression, the activation of NFκB pathway mediated by TNF-α is well described as a metastasis inductor. For that reason, TNF-α could be used as a prognostic marker in breast cancer [68,69,70]. Moreover, it was demonstrated that a strong relation between TNF-α levels and vascular endothelial growth factor D (VEGF-D) expression in samples of lymphatic metastasis from gallbladder cancer patients. The activation of ERK pathway mediated by TNF-α induces expression of VEGF-D, responsible for lymphangiogenesis and metastasis promotion [71].

The MDR phenotype may be acquired through cell-to-cell communication via MP, and growing evidence imply the MP role in cancer promotion by mediating the crosstalk between a tumor and its microenvironment [10,31,72,73]. Accordingly, we demonstrated that Pgp is carried by C1MP, albeit it could not be transferred to recipient non-tumor cells, presumably because of C1MP’s distinct biological properties (Figure 5). In fact, extracellular vesicles represent a heterogeneous population of vesicles which may cause dissimilar biological effects on recipient cells [72]. It has been shown that exosomes and MP derived from normal cells under an inflammatory context are competent in carrying TNF-α [22,74]. Exosomes could represent a refined form to regulate immune cellular responses by carrying active molecules [75,76,77,78]. In rheumatoid arthritis, exosomes secreted by fibroblasts contain the tmTNF-α form. The authors observed that long-term co-culture of activated CD4^+^ T cells plus exosomes caused a delay in activation-induced cell death in CD4^+^ T cells and a sustained proliferation for six days after stimulation [22]. Besides the role of extracellular vesicles in inflammatory disease, few articles showed extracellular vesicles carrying TNF family protein members in the cancer context [79,80]. Moreover, it was observed levels of human TNF-α mRNA in a murine cell line co-cultured with MP shed from a human osteosarcoma cell line [81]. However, we demonstrated for the first time a detectable TNF-α protein in MP derived from cancer cell lines (Figure 5B). Even though recipient cells do not clearly increase TNF-α proteins after co-culture with 3-1MP or C1MP (Figure 5D), both sTNF-α and tmTNF-α present in MP may interact with non-tumor recipient cells and induce the cellular responses demonstrated here, through activation of ERK phosphorylation and an increase of the cellular proliferation rate (Figure 8).

In this work, we present a cellular model to understand the role of TNF-α in a tumor MDR microenvironment. Considering the relation of TNF-α and the progression of different types of cancer, based on clinical data and the importance of Pgp in chemotherapy failure, we suggest that rTNF-α could support the MP release of cancer cells and spread Pgp to maintain an MDR phenotype. Since high TNF-α levels and Pgp expression are related to metastasis formation in different types of cancer, our results suggest that Pgp and TNF-α carried by MP could induce transformation of adjacent non-tumor cells. In conclusion, we propose that rTNF-α differentially regulates tumor resistance in sensitive and MDR cell lines supporting MP release, contributing to aberrant cell proliferation of non-tumor recipient cells.

## Supplementary Materials

The following are available online at https://www.mdpi.com/2073-4409/8/5/500/s1. Figure S1: Analysis of parental and MDR cells viability after treatment with different drugs. Figure S2: Efflux activity of Pgp in cancer cell lines. Figure S3: Microparticles derived from parental and MDR cell lines. Figure S4: Analysis of Pgp efflux activity by calcein-AM assay. Figure S5: Microparticles derived from parental and MDR cell lines.
