# Supplementary material for: TNF-α Modulates P-Glycoprotein Expression and Contributes to Cellular Proliferation via Extracellular Vesicles
Source: Cells. 2019 May 24;8(5):500. doi: 10.3390/cells8050500 (PMC6562596; doi:10.3390/cells8050500)
Supplement: Supplementary file 1 [file cells-08-00500-s001.zip › Supplementary Legends.docx]

**Supplementary 1.** Analysis of parental and MDR cells viability after treatment with different drugs. Cell viability determined by MTT assay after 24, 48 and 72 hours of treatment with cisplatin, doxorubicin or colchicine in KB-3-1 and KB-C1 cells. Data are means ± SD of three independent experiments. One-way ANOVA with Dunn’s posttest was used to analyze the MTT assay results*. * p < 0.05; ** p < 0.01; *** p < 0.005* indicates the statistical significance of data compared to non-treated cells.

**Supplementary 2.** Efflux activity of Pgp in cancer cell lines. Pgp efflux activity was determined by rhodamine 123 (Rho) assay in KB-3-1 and KB-C1 cells using flow cytometry. Empty black dashed histogram represents cellular autofluorescence; black histogram represents Rho staining and gray histogram represents Rho staining after CsA incubation.

**Supplementary 3.** Analysis of parental and MDR apoptosis-induced by rTNF-α treatment. Cell viability determined by Annexin-V/PI positive staining after 24 or 48 hours of rTNF-α treatment in different concentrations in KB-3-1 and KB-C1 cells.

**Supplementary 4.** Analysis of Pgp efflux activity by Calcein-AM assay**.** Efflux activity determined by Calcein-AM assay in KB-C1 cells after 24 hours of rTNF-α treatment, using flow cytometry. Empty black histogram represents cellular autofluorescence; black histogram represents Calcein-AM staining; empty gray histogram represents Calcein-AM staining after CsA incubation; dark gray and light grays histograms represent Calcein-AM staining after treatment with 10 or 15ng/mL rTNF-α, respectively. Dark gray dashed and light gray dashed histograms represent Calcein-AM staining after treatment with 10 or 15ng/mL rTNF-α and CsA incubation, respectively.

**Supplementary 5.** Microparticles derived from parental and MDR cell lines. Total count of MP derived from KB-3-1 and KB-C1 cells, determined by Annexin-V staining and size between 1 and 2µM, using flow cytometry.
