# Supplementary figures and images for: TNF-α Modulates P-Glycoprotein Expression and Contributes to Cellular Proliferation via Extracellular Vesicles
Source: Cells. 2019 May 24;8(5):500. doi: 10.3390/cells8050500 (PMC6562596; doi:10.3390/cells8050500)

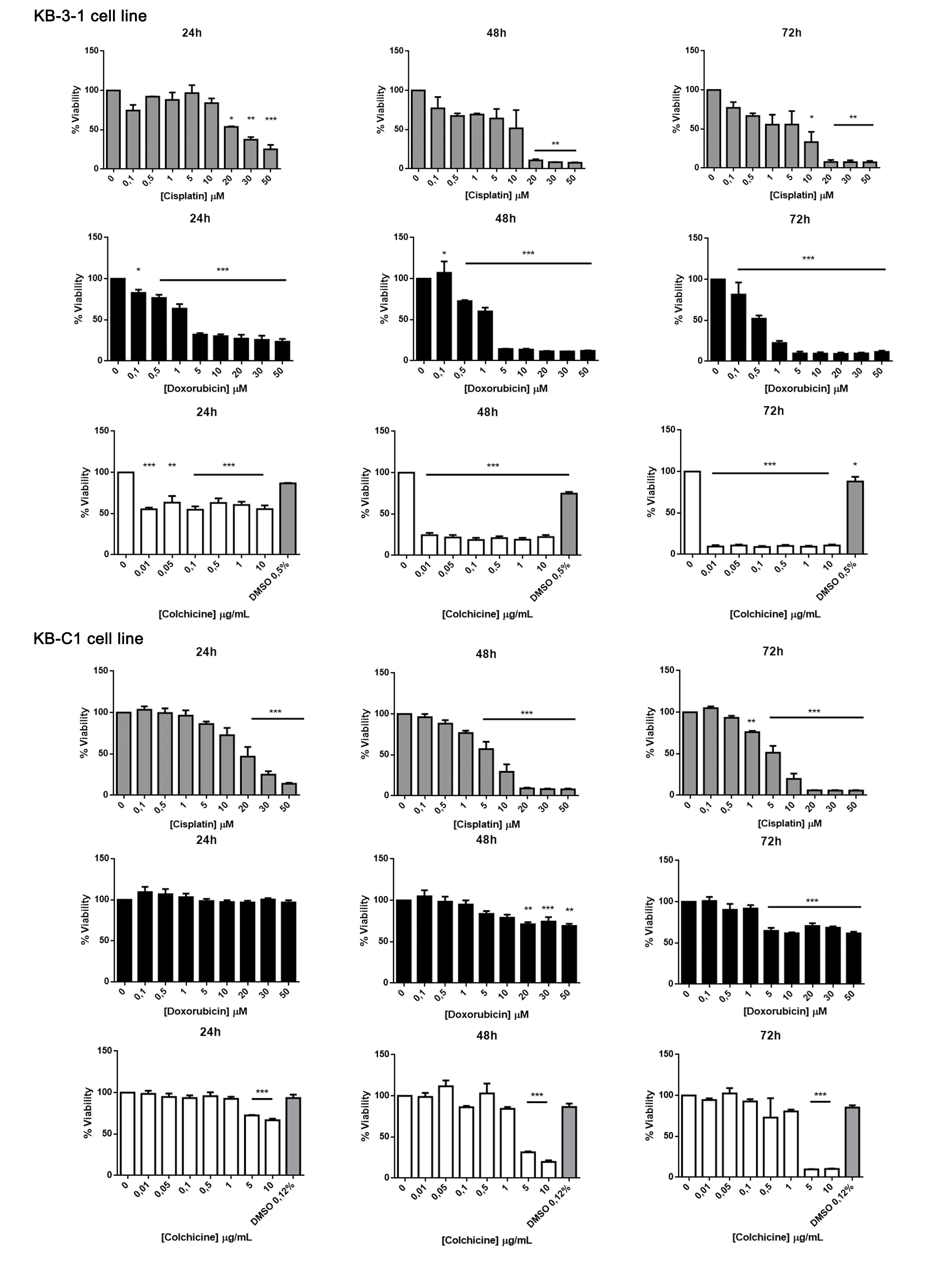

Supplement: Supplementary file 1 [file cells-08-00500-s001.zip › Fig_S1.tif]

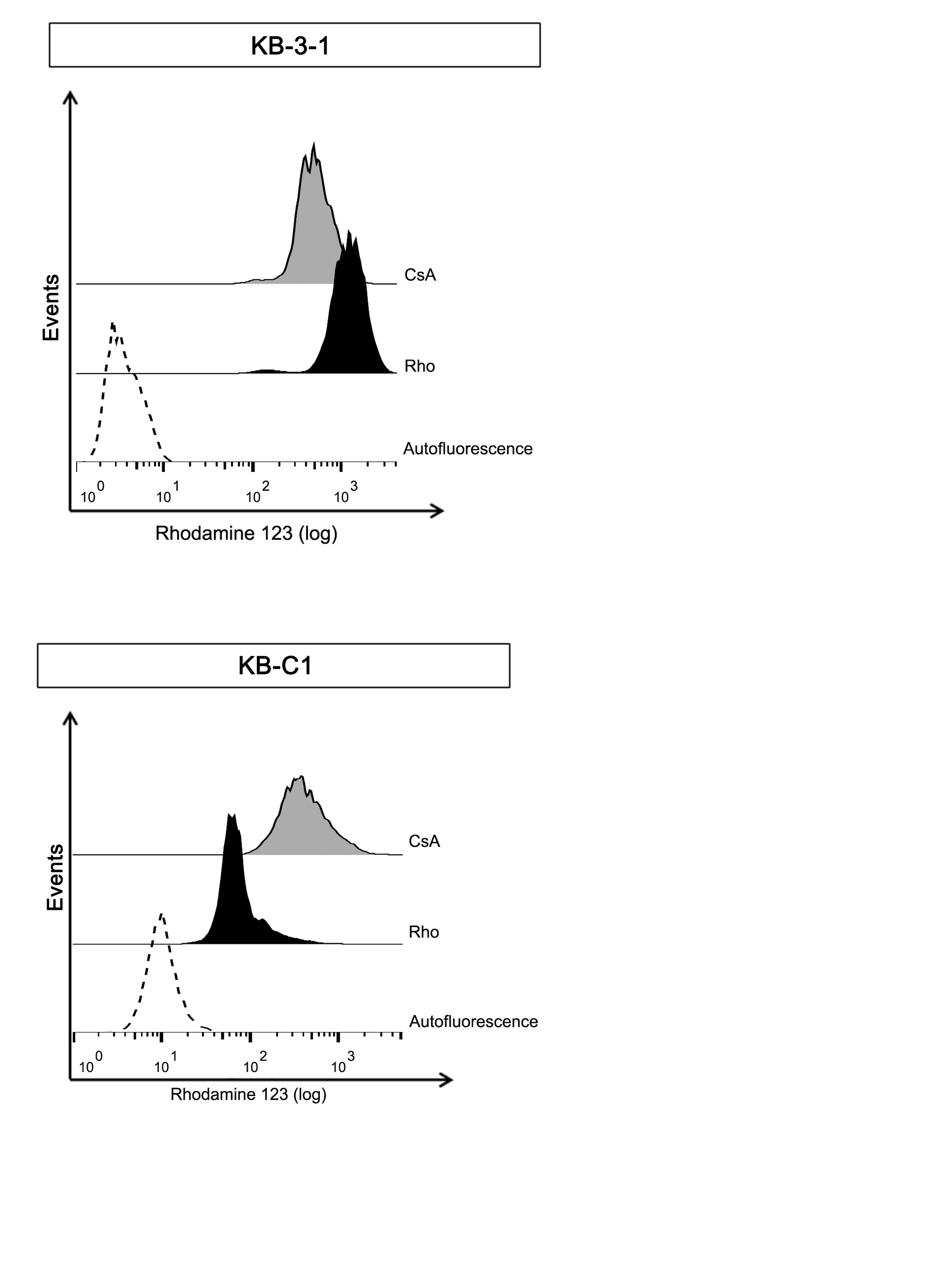

Supplement: Supplementary file 1 [file cells-08-00500-s001.zip › Fig_S2.tif]

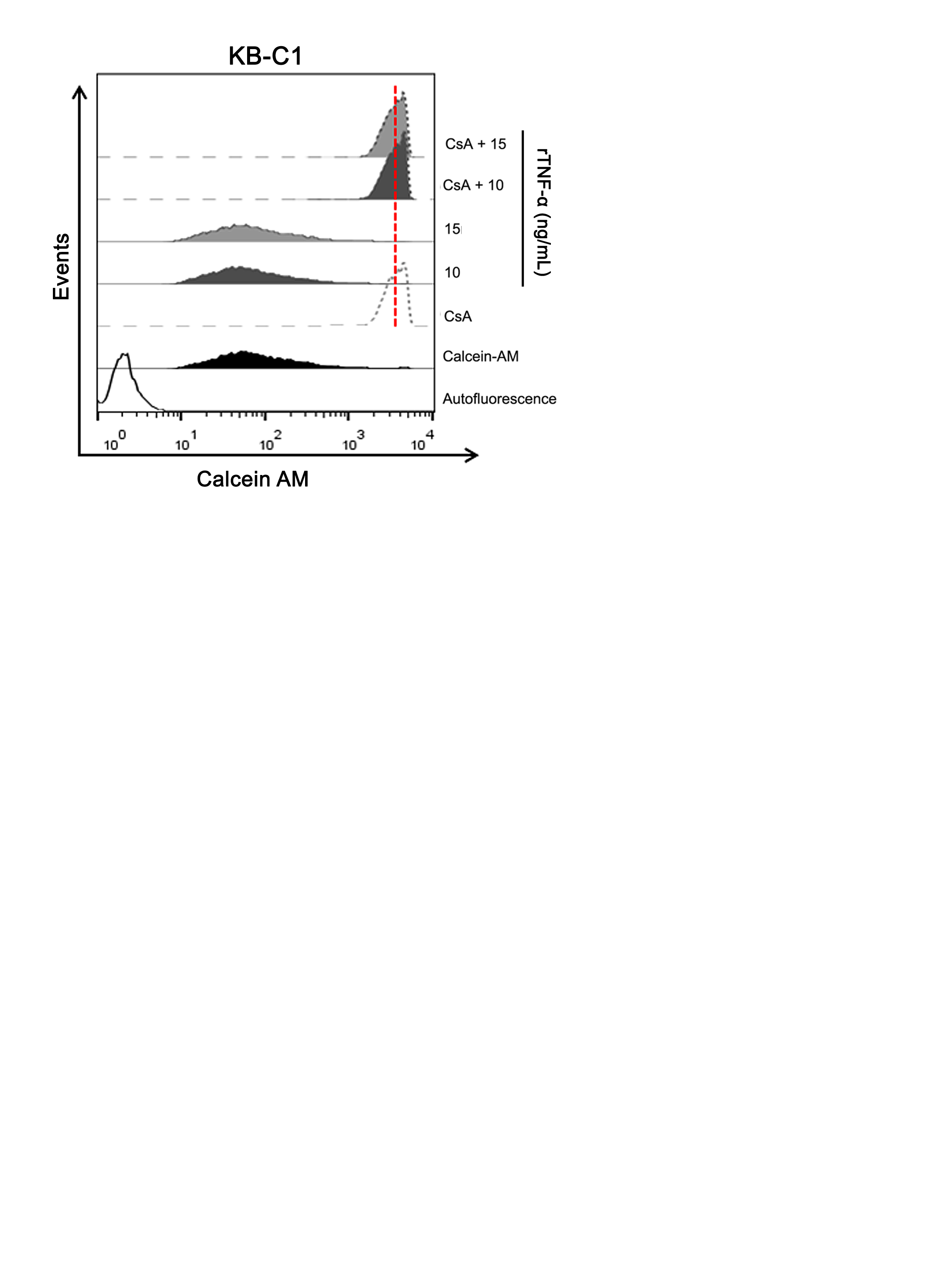

Supplement: Supplementary file 1 [file cells-08-00500-s001.zip › Fig_S4.tif]

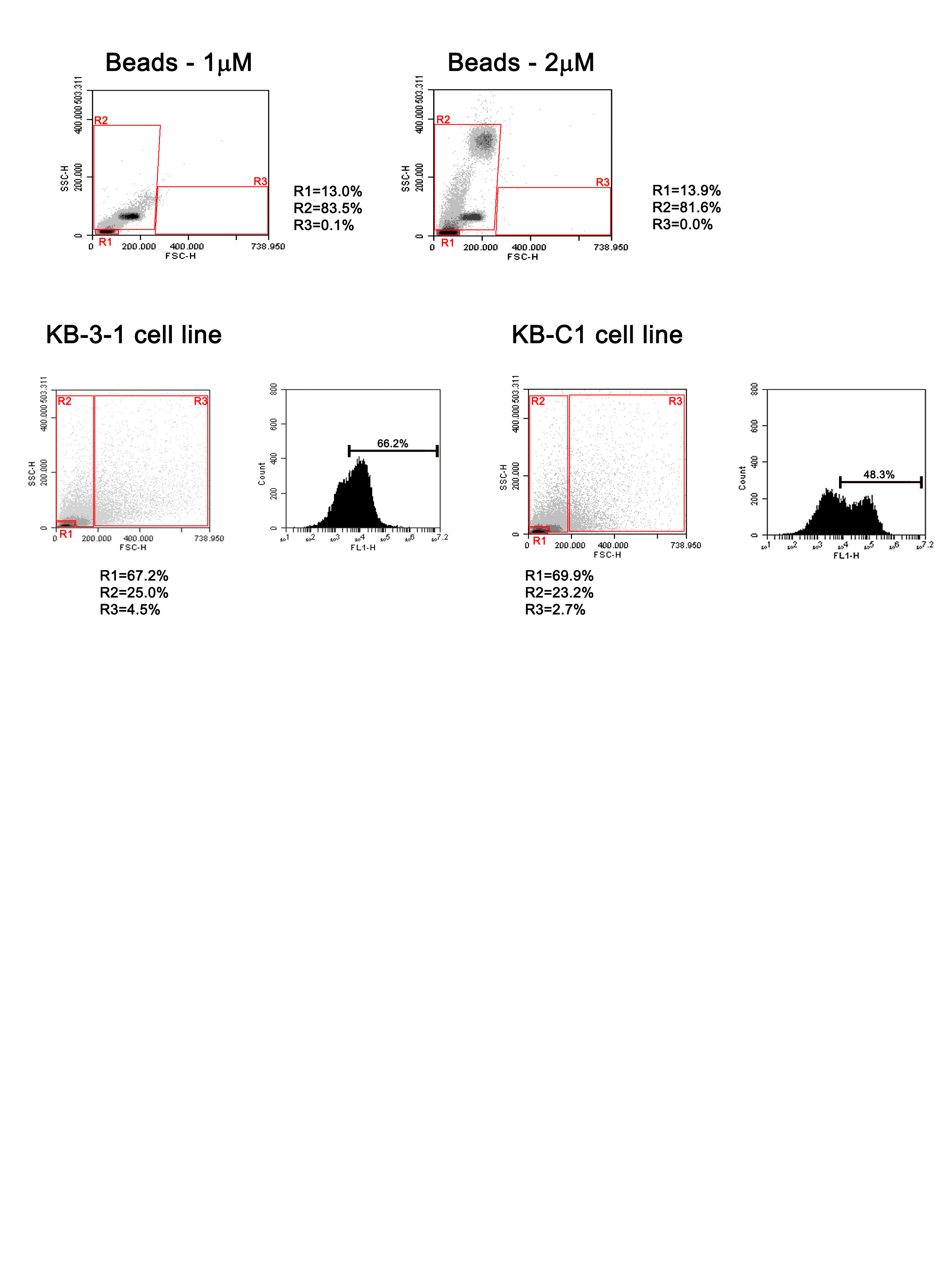

Supplement: Supplementary file 1 [file cells-08-00500-s001.zip › Fig_S5.tif]
